# Association of personality traits and traffic accident involvement: a multicenter case-control study in Iran

**Published:** 2025-07

**Authors:** Reza Fereidooni, Amin Reza Masoumi, Saeed Kargar Soleimanabad, Mina Sadeghi, Tahereh Ghahramani, Zivar Amani, Seyyed Hamidreza Ayatizadeh, Yaser Sarikhani, Mohammad-Rafi Bazrafshan, Seyed Taghi Heydari, Kamran Bagheri Lankarani

**Affiliations:** ^*a*^ Health Policy Research Center, Institute of Health, Shiraz University of Medical Sciences, Shiraz, Iran.; ^*b*^ Trauma Research Center, Shahid Rajaee (Emtiaz) Trauma Hospital, Shiraz University of Medical Sciences, Shiraz, Iran.; ^*c*^ Research Center for Social Determinants of Health, Jahrom University of Medical Sciences, Jahrom, Iran.; ^*d*^ Department of Nursing, School of Nursing, Larestan University of Medical Sciences, Larestan, Iran.

**Keywords:** Personality, Personality tests, Traits, NEO personality inventory, NEO-FFI, Case-control study, Risky driving

## Abstract

**Background:**

Driver-associated factors are significant contributors to road traffic accidents. Conversely, personality traits are the characteristics and qualities that define an individual’s consistent patterns of thoughts, feelings, and behaviors. Driving behavior is influenced by a variety of factors. We hypothesized that different personality traits may affect driving behavior. This study aimed to investigate the relationship between various personality traits and involvement in accidents.

**Methods:**

Drivers with a history of accidents resulting in injuries for which they were at fault were classified as cases, and drivers without a history of an accident in the past year were considered as controls. We assessed the Big Five personality traits among all participants using the NEO Five-Factor Inventory (NEO-FFI) test. Additionally, we collected data on potential determinants of high-risk driving, including age, marital status, education, alcohol consumption, smoking, psychiatric disorders, self-assessment of driving skills, and substance abuse. The NEO-FFI test scores were compared between cases and controls. We employed Partitioning Around Medoids (PAM) to create clusters for each personality trait. Logistic regression was utilized to examine the association between the independent variables and the clusters of personality traits, adjusting for potential confounders such as age, marital status, and education level.

**Results:**

A total of 662 participants, comprising 393 cases and 269 controls, were recruited for the study. The mean score for neuroticism was significantly higher in the case group, while the mean scores for extroversion, agreeableness, and conscientiousness were substantially lower. The mean score for openness to experience did not show a significant difference. The Personality Assessment Model (PAM) identified two clusters for all personality traits, labeled as high and low. In the logistic regression model, high levels of neuroticism (aOR: 2.75, 95%CI: 1.69-4.45) and low levels of conscientiousness (aOR: 0.50, 95%CI: 0.30-0.84) were associated with an increased likelihood of being involved in a car accident.

**Conclusions:**

Drivers involved in severe accidents tended to exhibit higher levels of neuroticism and lower levels of extraversion, conscientiousness, and agreeableness, as measured by the NEO-FFI. Regression analysis revealed that elevated neuroticism and diminished conscientiousness were significantly associated with high-risk driving behaviors. Although assessing personality traits can aid in predicting risky driving, this association is not definitive, and caution should be exercised when generalizing these findings.

## Introduction

Road traffic accidents are among the leading causes of mortality and morbidity worldwide, claiming over 1.2 million lives and resulting in 50 million injuries each year.^1,2^ Numerous factors contribute to traffic accidents, with driver-related factors regarded as the most significant contributors.^3,4^ Risky driving behaviors, such as distracted driving, speeding, and driving under the influence, have long been recognized as critical issues.^5^ Recently, there has been increased focus on the determinants of these risky driving behaviors.^6-9^ One well-known determinant is the psychological factors, including the cognition and personality traits of drivers.^10,11^

Personality encompasses the characteristic sets of behaviors, cognitions, and emotional patterns shaped by both biological and environmental factors, and it evolves over time.^12^ A growing body of evidence has explored the relationship between personality and driving behavior.^13-16^ One of the most widely utilized instruments for assessing personality traits is the Revised NEO Personality Inventory (NEO-PI-R) and its short form NEO Five-Factor Inventory (NEO-FFI).^17^ These assessments evaluate the five dimensions of personality, commonly referred to as the Big Five: neuroticism, extraversion, openness to experience, conscientiousness, and agreeableness. Additionally, five facets have been identified for each of these personality traits.^18^

The association between the Big Five personality traits and risky driving or road traffic accidents has been demonstrated in several studies.^19-21^ A study conducted by Lajunen et al.^22^ revealed that countries with high extraversion scores experienced higher traffic accident mortality rates compared to those with moderate or low extraversion scores. Numerous studies have explored the relationship between specific facets of personality traits and risky driving behaviors. For instance, depression and anger, both facets of neuroticism, as well as sensation seeking, a facet of extraversion, are linked to risky driving behaviors.^23-25^ In a meta-analysis of 22 studies, it was found that low agreeableness, high neuroticism, sensation seeking, and driving anger were significantly associated with risky driving behaviors. However, no significant correlations were identified for extraversion, conscientiousness, and openness to experience.^20^

In Iran, studies have emerged to assess the relationship between personality traits and driving behavior. However, most of these studies have focused on specific traits such as anxiety,^26^ sensation seeking, and normlessness.^27^ Additionally, Type A/B personality was also compared among high-risk and low-risk drivers in Iran.^28^ Two studies evaluated the Big Five personality traits using the NEO Personality Inventory; however, both studies exclusively recruited heavy vehicle drivers (bus or truck).^29,30^ Alavi et al.^29^ employed prospective methods to monitor crashes over two years, and found that only neuroticism was associated with crash probability. Ghanavati et al.^30^ utilized the Manchester Driving Behavior Questionnaire and reported a significant positive correlation for the dimension of neuroticism. However other personality trait dimensions exhibited a significant negative relationship with unsafe driving behaviors.

Another study conducted in Iran demonstrated a positive correlation between neuroticism and a negative correlation between agreeableness and extraversion with risky driving behavior. This study also employed convenience sampling methods and utilized the Manchester Driving Behavior Questionnaires to identify high-risk and low-risk drivers.^31^ Additionally, one study explored the relationship between the Big Five personality traits and mobile phone usage while driving, which is considered a form of risky driving behavior. It concluded that extraversion, agreeableness, and conscientiousness were associated with a higher prevalence of mobile phones while driving.^32^ The current study differs from previous studies in Iran by encompassing a more diverse population and incorporating traffic accident involvement as a criterion for the case and control groups.

This study investigates the relationship between personality traits and involvement in traffic accidents where the individual is at fault. By conducting a multicenter study across two distinct geographical regions in Iran, our research encompasses a diverse population characterized by various cultural, environmental, and socio-economic factors. This diversity is crucial, as personality expression and driving behaviors can vary significantly across different contexts, potentially influencing the likelihood of accidents. Consequently, our study addresses a significant gap in the existing literature by providing a comprehensive understanding of the relationship between personality traits and traffic accidents in varied settings. This enhances the applicability of our findings for targeted interventions and policy development. 

## Methods 

In this case-control study conducted in 2023, two major cities in Iran were selected as study locations: Shiraz, the capital of Fars Province, and Sari, the capital of Mazandaran Province. These cities were chosen to represent distinct geographic and cultural contexts. Shiraz is the fifth most populous city in Iran, located in the southwestern of the country. As the capital of Fars Province, Shiraz is renowned for its dry climate, challenging mountainous road conditions, and rural roads that wind through gorges. Consequently, it consistently ranks high in traffic casualties in Iran. Sari is a city located in the Central District of Sari County, in Mazandaran Province. It serves as the capital of the district, the county, and the province. Sari is the largest and most populous city in Mazandaran. In contrast to Shiraz, Mazandaran Province, known for its high precipitation, dense forests, steep roads, and numerous closely situated cities, has relatively lower casualty rates. By selecting these provinces, the study aimed to capture a diverse range of road conditions and driver characteristics prevalent in various regions of Iran.^33^ This study adheres to the ethical norms and standards outlined in the Declaration of Helsinki and received approval from the ethics committee of Shiraz University of Medical Sciences under the code IR.SUMS.REC.1399.931.

**Sampling and data collection**

Sample size determination was based on the findings of a similar study conducted in Iran. The study reported NEO neuroticism scores of 29.85 ± 10.24 for drivers involved in accidents and 31.92 ± 6.52 for drivers without accident involvement.^34^ To achieve a statistical power of 0.9 (90%) with an alpha level of 0.01 (1% significance level), we employed a two-sample comparison formula. The calculation indicated that a minimum sample size of 90 participants would be required, with 45 participants in the case group and 45 in the control group.

A total of 662 drivers participated in this study, including 393 with a history of accidents resulting in injuries and 269 without such history, from two cities in Iran. It has been demonstrated that involvement in car accidents, as well as the severity of these accidents, is linked to high-risk driving behavior.^1,35,36^ We considered drivers with a history of being at fault in accidents resulting in injury to be an acceptable representation of high-risk drivers. Shahid Rajaei (Emtiaz) Hospital, a Level 1 trauma center in Shiraz, and Imam Hospital in Sari were selected for this study because they serve as the primary trauma centers in their respective provinces. As a control group, 269 randomly selected participants with no history of accidents resulting in injuries were recruited from main streets, parks, and malls in various districts of Shiraz and Sari at different times, utilizing the convenience sampling method. The control group was selected using convenience sampling due to practical limitations, such as restricted access to a larger population and the lack of a sampling frame. While this method facilitates quick data collection, it inherently poses a risk of introducing biases. Convenience sampling may result in a control group that does not accurately reflect the target population, potentially affecting the generalizability of the study's results. Specifically, this technique can introduce selection bias, as participants who are easily accessible or willing to participate may systematically differ from those who are not, in terms of demographics, health status, or other relevant characteristics.

As inclusion criteria, we required informed consent, possession of a valid driver's license, and an age of over 18 years. An additional criterion for the case group was the presence of a vigilant level of consciousness, enabling participants to respond to the questions. For the case group, individuals with a history of at least one traffic accident in the past year that resulted in injuries and for which they were at fault were included. In contrast, the control group consisted of individuals with no history of any traffic accidents in the past year, which served as an additional inclusion criterion. Exclusion criteria included a reluctance to cooperate at any stage of the research and a low level of consciousness in the patients. We also gathered data on the possible determinants of high-risk driving, including age, gender, type of license, marital status, driving experience, education, self-assessment of driving skills, psychiatric disorders, alcohol consumption, smoking, and substance abuse. The psychiatric disorders of interest included depression, bipolar disorder, anxiety spectrum disorders, and psychotic spectrum disorders, all of which were confirmed by a psychiatrist based on the participants' self-reports.

We utilized the short-form NEO Personality Inventory, known as the NEO Five-Factor Inventory (NEO-FFI), developed by Costa and McCrae^37^ for data collection. The five personality traits measured by this test are neuroticism, extraversion, openness to experience, conscientiousness, and agreeableness. This scale consists of 60 Likert statements, with 12 items corresponding to each trait. Each statement is assigned an integer score ranging from 0 to 4, resulting in a total score for each trait that can range from 0 to 48. The validity and reliability of the NEO-FFI questionnaire have been established by Costa and McCrae.^17^ The Persian version of the NEO Personality Inventory was standardized by Garousi Farshi et al., and the correlation coefficients for the five main dimensions were reported to range from 0.56 to 0.87.^38^ In addition, we collected demographic variables and other data that may be correlated with risky driving behavior, including age, gender, marital status, education, a history of professionally diagnosed psychiatric disorders, alcohol use, substance abuse, years of driving experience, and self-reported driving skills.

The question concerning driving skills was first introduced by Svenson in 1981^39^ It asked participants to evaluate their skills in comparison to a broader community across five categories: very low, low, moderate, high, and very high.

Other data, including the history of psychiatric disorders, substance abuse, and previous accidents, relied on self-reporting. It is important to note that, due to limitations in data availability, we were unable to cross-check the accident and disease databases to validate the self-reported information provided by the participants. This was primarily due to the absence of a comprehensive disease database in Iran, coupled with the secretive and incomplete nature of the accident database. These limitations hindered our ability to independently verify the reported accident histories and psychiatric disorders using official records.

**Statistical analysis**

The demographic characteristics of these two groups were collected and presented as descriptive statistics. An independent t-test was employed to compare the mean scores in neuroticism, extraversion, openness to experience, conscientiousness, and agreeableness between the case and control groups.

Given the lack of predefined reference values for distinguishing high or low-level subcategories of personality traits in the NEO-FFI, we utilized the partitioning around medoids (PAM) technique to generate clusters of personality traits.^40^ The PAM algorithm identifies k representative items, known as medoids, within the dataset. It assigns each item to its nearest medoid, intending to minimize dissimilarities between the objects in a cluster and their center. PAM is recognized for its robustness against outliers, making it a reliable alternative to traditional k-means clustering. PAM was applied to each of the five personality traits. The optimal number of clusters was determined using internal clustering validity indices, specifically the Silhouette and Calinski-Harabasz indices, along with stability measures, including the average proportion of non-overlap (APN) and the average distance between means (ADM). Internal clustering validity indices with maximum values indicate clusters that exhibit a high level of separation and compactness. Stability measures with minimum values suggest that the clusters are stable and will not be significantly affected by the removal of variables. Finally, two clusters were selected for each trait.

Multiple logistic regression was employed to assess the relationship between clusters of personality traits and involvement in a severe accident while at fault, compared to individuals with no history of car accidents over the past year. The regression model accounted for various determinants of risky driving, including age, marital status, education level, type of license, alcohol consumption, self-assessment of driving skills, smoking habits, psychiatric disorders, and substance abuse. Female drivers were significantly underrepresented; therefore, the gender variable was excluded from the model. The driving experience was also overlooked due to a high degree of collinearity with age. To achieve a more balanced representation and minimize the impact of an inflated regression model when preparing self-reported driving skills, the initial five options were categorized as "Low to Moderate," "Moderate to High," and "High to Very High." This approach aims to create more equitable subgroups. To gain a comprehensive understanding of how personality traits influence one another and are affected by various measured factors, we conducted a multivariable regression analysis. Adjusted odds ratios (aOR) and their 95% confidence intervals (95% CI) are reported. A p-value of less than 0.05 was considered statistically significant. Internal clustering validity indices, stability measures, and PAM were executed using R package version 4.0.5 and packages "MixSim", "cluster", "e1071", "mclust", "clusteval", "ClustCrit", and "clValid". Other analyses were done using Stata Statistical Software: Release 14. College Station, TX: StataCorp LP.

## Results

A total of 662 drivers participated in this study, comprising 393 individuals with a history of accidents resulting in injuries and 269 without such a history, from two cities in Iran. Two hundred-seven cases and 156 controls were from Shiraz, while 186 cases and 113 controls were from Sari. The response rate among cases was 84.5%, while the response rate among controls was 72.7%. Male drivers accounted for 94.41% of all participants.

**Figure 1:**
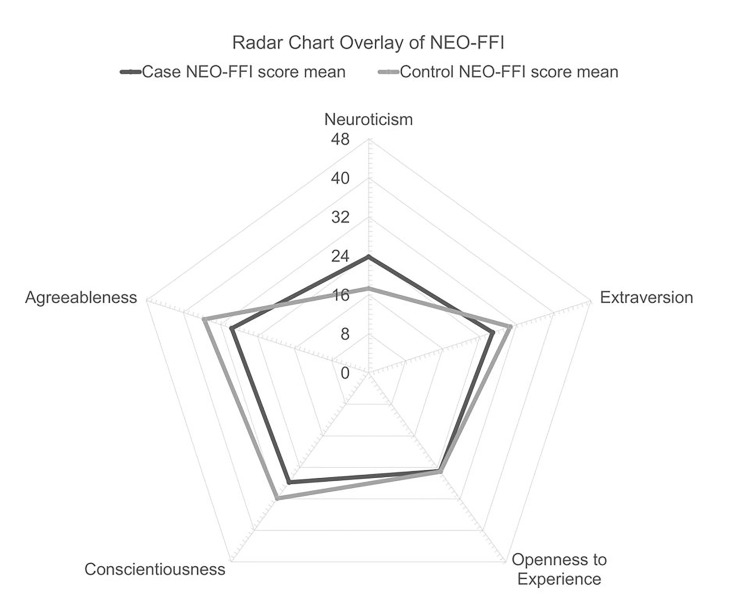
Radar chart of NEO-FFI personality trait scores in case and control group

Applying the t-test, the mean score for neuroticism was significantly higher in the case group, while the mean scores for extroversion, conscientiousness, and agreeableness were significantly lower than those in the control group. The mean score for openness to experience did not differ significantly between the two groups (Table 1).Figure 1 represents a radar chart overlay of the mean scores for personality traits in both the case and control groups.

**Table 1:** Mean scores of the NEO-FFI test for each personality trait and the results of the t-test comparing case and control groups.

Personality traits	Case (n=393)		Control (n=269)		P value
	NEO FFI score mean	Standard deviation	NEO-FFI score means	Standard deviation	
**Neuroticism**	23.81	5.95	17.31	7.55	**<0.001**
**Extraversion**	26.78	7.94	30.58	8.53	**<0.001**
**Openness to experience**	24.96	5.21	25.10	4.40	**<728**
**Conscientiousness**	27.78	7.79	31.90	8.26	**<0.001**
**Agreeableness**	29.55	8.31	35.52	8.02	**<0.001**

Internal clustering validity indices and stability measures indicated that the two clusters were most appropriate for all traits. The two clusters were labeled "Low" and "High," and the PAM algorithm assigned the observations to each cluster. Table 2 presents the NEO-FFI scores for the trait clusters, along with the number of participants in each cluster. Using the chi-square test, the analysis revealed that the prevalence of high openness did not differ significantly between the two groups. However, neuroticism was found to be higher, while extroversion, agreeableness, and conscientiousness were lower in the case group. Applying the logistic regression model and adjusting for potential confounders, neuroticism was positively associated with being at fault in a car accident (aOR: 2.75, 95% CI: 1.69-4.45, p-value <0.001). In contrast, the association with conscientiousness was negative (aOR: 0.50, 95% CI: 0.30-0.84, p-value: 0.009).

**Table 2:** Characteristics of each cluster of personality traits.

	Trait cluster	NEO-FFI score range (min-max)	NEO-FFI score means	Control, n (%) (n=269)	Case, n (%) (n=393)	Total (n=662)	P value
**Neuroticism**	Low	3-20	14.47	166 (61.7)	117 (29.8)	283 (42.8)	<0.001
	High	21-40	26.16	103 (38.3)	276 (70.2)	379 (57.3)	
**Extraversion**	Low	0-28	21.94	108 (40.2)	234 (59.5)	342 (51.7)	<0.001
	High	29-48	35.10	161 (59.9)	159 (40.5)	320 (48.3)	
**Openness to experience**	Low	4-25	21.40	139 (51.7)	215 (54.7)	354 (53.5)	0.442
	High	26-39	29.16	130 (48.3)	178 (45.3)	308 (46.5)	
**Conscientiousness**	Low	0-29	23.32	113 (42.0)	238 (60.6)	351 (53.0)	<0.001
	High	30-48	36.33	156 (58.0)	155 (39.4)	311 (47.0)	
**Agreeableness**	Low	0-33	25.89	103 (38.3)	276 (70.2)	379 (57.3)	<0.001
	High	34-48	40.07	166 (61.7)	117 (29.8)	283 (42.8)	

When examining additional factors, a greater number of professional license types were linked to a lower incidence of accident involvement, whereas higher self-reported driving skills correlated with an increased likelihood of being involved in an accident. Age and education did not show a significant association with the outcome. However, smoking and alcohol consumption were significantly related to accident involvement (Table 3).

**Table 3:** Factors associated with being at fault in accidents leading to injury.

Variables		Adjusted odds ratio	95% Confidence interval		P value
			Lower	Upper	
**Big Five personality traits**	High neuroticism	2.73	1.64	4.53	<0.001
	High extraversion	1.51	0.89	2.57	0.127
	High openness to experience	1.23	0.77	1.98	0.382
	High agreeableness	1.48	0.85	2.57	0.161
	High conscientiousness	0.56	0.33	0.95	0.033
**License type ^a^**	Motorcycle	Ref.	-	-	-
	B2	2.73	0.20	37.09	0.448
	B1	0.06	0.00	1.03	0.053
	P1	0.01	0.00	0.24	0.005
**Education level**	Less than a high school diploma	Ref.	-	-	-
	High school diploma	0.36	0.16	0.83	0.018
	Associate’s degree	0.94	0.35	2.48	0.897
	Bachelor’s degree	0.45	0.19	1.06	0.068
	Master’s degree or higher	0.63	0.24	1.65	0.346
**Marital status**	Single	Ref.	-	-	-
	Married	0.85	0.39	1.85	0.690
	Divorced or widowed	1.47	0.52	4.12	0.467
**Self-reported driving skill**	Low to moderate	Ref.	-	-	-
	Moderate to high	2.53	1.35	4.77	0.004
	High to very high	3.58	1.82	7.07	<0.001
**Age group**	<30	Ref.	-	-	-
	31-40	1.41	0.62	3.21	0.416
	41-50	0.94	0.36	2.46	0.896
	>50	1.00	0.38	2.61	0.997
Alcohol use		4.55	2.24	9.21	0<.001
History of psychiatric disorder ^b^		3.05	1.09	8.55	0.034
Smoking (waterpipe/hookah or cigarette)		5.45	3.29	9.02	<0.001

a Motorcycle: Motorcycles/Motorized tricycles, B1: Motor vehicles with a seating capacity for not more than 9 passengers and vehicle up to 3500 kg Gross Vehicle Weight (private), B2: Motor vehicles with a seating capacity up to 26 passengers and vehicle up to 6000 kg Gross Vehicle Weight (transportation), P1: Buses and trucks with a capacity over 6000 kg Gross Vehicle Weight b Psychiatric disorders included depression, bipolar disorder, anxiety spectrum disorder, and psychotic spectrum disorder confirmed by a psychiatrist

In Table S1, we present the associations between clusters of personality traits and various other traits and study variables for all participants. Neuroticism is significantly associated with an increased odds of having a history of psychiatric disorders and smoking. Moreover, individuals with high levels of neuroticism tend to exhibit lower levels of agreeableness. Extroversion, in contrast, demonstrated positive correlations with agreeableness and conscientiousness, while exhibiting an inverse relationship with alcohol consumption and smoking. Openness to experience did not show significant associations with other personality traits. Younger drivers (under 30 years old) tended to exhibit higher levels of openness compared to their older counterparts. Notably, agreeableness and conscientiousness exhibited a strong positive relationship. However, agreeableness was associated with an increased likelihood of having a history of psychiatric disorders and smoking. In contrast, higher conscientiousness was linked to a reduced likelihood of alcohol consumption, smoking, or having a psychiatric disorder.

## Discussion

This case-control study aims to assess the association between personality traits, as measured by the NEO Personality Inventory, and risky driving behavior. The study recruits a group of drivers who were at fault in accidents as the case group. Initially, we conducted partitioning around the medoids to identify clusters corresponding to each personality trait. Consequently, the participants' personality traits were categorized into high-risk and low-risk groups. The results of this study confirm that a high level of neuroticism and a low level of agreeableness are significant predictors of risky driving behavior.

In earlier research, the relationship between neuroticism and involvement in traffic accidents was unclear and often inconsistent.^22^ However, recent studies, particularly those that adjust for multiple confounders and utilize the NEO-FFI, widely accept that personality traits positively correlate with aggressive driving and involvement in accidents.^41,42^ Alavi et al.^29^ found that only neuroticism was associated with traffic crashes, while other personality traits were not. Neuroticism is characterized by negative emotions, including anger, anxiety, self-consciousness, irritability, emotional instability, and depression.^43^ It is postulated that the correlation between risky driving and neuroticism is mediated by anger, a facet of neuroticism.^42^ One study has found that higher levels of neuroticism have a direct effect on the number of crashes and driving anger, as measured by the Driving Anger Expression Inventory.^44^ Neuroticism is also linked to substance abuse and various mental disorders, such as anxiety disorders, major depressive disorder, bipolar disorder, psychosis, and schizophrenia.^45,46^ These factors are determinants of risky driving behavior; however, we attempted to adjust the analysis for substance abuse and certain psychiatric disorders.^9^ Bouts of depressive moods and anxiety occur more frequently in individuals with high levels of neuroticism when confronted with stressors.^43^ These two dispositions are associated with involvement in collisions.^47^

Some studies have indicated that both conscientiousness and agreeableness are associated with a higher likelihood of engaging in risky driving behavior.^48-50^ Several studies have linked lower levels of agreeableness with risky driving behavior. In an analysis of 308 drivers, Dahlen et al.^51^ found that drivers with low levels of agreeableness were more likely to demonstrate aggressive driving behavior. Habibifar et al.^52^ found that conscientiousness and agreeableness are negatively correlated with unsafe driving styles, such as inattentiveness, anxiety, and nervousness, but positively associated with cautious driving. While this finding was replicated in our study, the results of the multiple logistic regression indicated a significant relationship only for conscientiousness.

The correlation between openness to experience and risky driving behavior appears to be very minimal. A meta-analysis revealed no significant relationship between risky driving behavior and openness.^20^ Benfield et al.^49^ found that more aggressive driving is associated with lower levels of openness, while Taubman et al.^50^ discovered that openness is positively correlated with a more cautious driving style. Wang et al.^48^ found that a risky style was positively predicted by openness, whereas an angry and high-velocity style was inversely associated with openness. In conclusion, the significance of these findings is less clear, and our study did not identify a significant relationship between openness to experience and accident involvement.

Some aspects of extroversion include a tendency to be active, daring, energetic, and sensation-seeking.^50^ Generally, maladaptive driving styles are believed to have a positive correlation with extroversion.^48^ Sensation seeking, a facet of extroversion, is correlated with risky driving.^20^ Extroversion itself is also suggested to be positively and significantly associated with risky driving behavior.^20^ In our study, contrary to previous reports, we found that the case group exhibited a lower extroversion score compared to the control group. However, it is important to note that when categorized into high and low clusters and after accounting for confounding variables, the difference was not statistically significant. The absence of a relationship between extroversion and traffic accidents or high-risk driving is not unprecedented, particularly in Iran.^29-31^ This indicates that extroversion as a broad personality trait may not effectively predict driving behavior patterns. Instead, more specific facets or behaviors associated with this trait may exhibit a stronger correlation.

Agreeableness reflects how individuals interact with and treat others. Those with high levels of agreeableness are characterized by selflessness, empathy, and a willingness to assist others, often with the expectation of receiving similar treatment in return. Conversely, individuals with low agreeableness tend to exhibit antagonistic, selfish, and manipulative tendencies.^50,53^ Given this definition, a negative correlation between agreeableness and risky driving behavior is anticipated, as demonstrated in several previous studies.^20,21,49^ Similarly, in our study, drivers involved in accidents exhibited a significantly lower agreeableness score; however, the multiple logistic regression did not reveal a significant association. Additionally, the observation of higher odds of smoking, psychiatric disorders, and alcohol use among individuals with high agreeableness was unexpected, as existing literature suggests the contrary.^54^

Conscientiousness is a trait characterized by being cautious, organized, and responsible. It follows that individuals who exhibit this pattern of behavior tend to drive more carefully.^50^ As opposed to neuroticism, individuals with higher levels of conscientiousness exhibit a lower tendency to engage in health-threatening behaviors such as smoking, drug use, and reckless driving.^46,55^ This speculation is supported by the findings of this study. Despite the results of the logistic regression presented in Table 3, which do not suggest that conscientiousness and neuroticism are opposing traits, the differences in accident involvement, smoking, alcohol consumption, and psychiatric disorders highlight this issue.

The study's findings have significant implications for public health and traffic safety. Identifying personality traits as risk factors aids in the development of targeted interventions, early identification, and prevention efforts. This knowledge guides screening and assessment procedures, informs the implementation of interventions, and enables specific approaches to address individual needs and characteristics. Examples of interventions designed to promote safer driving practices based on personality traits include targeted education and training programs,^56^ social norming strategies,^56,57^ personalized feedback delivered through technology^58^ or coaching.^59^ driving license reforms,^29^ and incentive-based programs.^60^ While implementing psychological profiling and personalized interventions for drivers may pose challenges regarding feasibility and practicality, there is value in adopting a multifaceted approach. Recognizing that individuals may respond differently to interventions based on their personality traits is essential for tailoring these interventions to meet individual needs and characteristics. Personalized approaches enhance the likelihood of successful outcomes by addressing the unique challenges and motivations of each person. 

In Iran, social norms and peer influence significantly impact behavior. The tendency to conform to group behaviors, such as speeding, aggressive overtaking, or disregarding traffic regulations, can overshadow individual personality traits like conscientiousness and agreeableness. Drivers may feel compelled to engage in riskier behaviors if such actions are viewed as acceptable or even commendable within their social groups.^61^ This culture, driven by peer influence, can enhance traits such as thrill-seeking and impulsivity, making them more apparent in actual driving behavior. Furthermore, a common belief holds that arbitrary or inconsistent law enforcement affects driving behavior. When drivers perceive that traffic laws are not enforced fairly or consistently, individuals with personality traits such as low agreeableness or high antisocial tendencies may be more inclined to violate the rules, believing that the chances of facing punishment are minimal.^61,62^

In future studies, it would be advantageous to select cases and controls more carefully, utilizing self-reporting tools such as driving behavior questionnaires, in conjunction with objective measures like the number of road traffic accidents and the frequency and types of traffic violations.

**Limitations**

Female drivers were significantly underrepresented in this study; therefore, the gender variable was excluded from the logistic regression model. This indicates that risky driving behaviors are more common among males, who are also involved in more severe accidents, consistent with findings from previous research.^6^ The respondents were asked whether they smoked, consumed alcohol, or used drugs in general, regardless of whether they did so while driving. Research has demonstrated that individuals who engage in these behaviors are more likely to be high-risk drivers, even in the absence of concurrent use while driving.^63^ This may not correlate well with risky driving behavior. However, asking participants whether they drive under the influence of alcohol or while smoking may not yield accurate responses due to social desirability bias, fear of judgment or legal repercussions, and a desire to portray themselves in a favorable light. Generally, the self-report method is subjective and depends on the honesty of participants, which can be affected by various factors, resulting in potential underreporting or misrepresentation of behaviors.

One significant limitation of our study is the sample size, which may affect the generalizability and statistical power of our findings. Caution should be exercised when interpreting the results, as the smaller sample size increases the likelihood of random variation and may not adequately represent the broader population. Future studies involving larger and more diverse samples are necessary to validate and expand our findings. Additionally, the use of convenience sampling for the control group introduces the possibility of selection bias. Although efforts were made to match the control group based on province of residence, age, and sex, it is important to acknowledge that convenience sampling may not fully represent the broader population of interest. Future studies that utilize more representative sampling methods would improve the external validity of the findings. Furthermore, although this is a multicenter study, the results may not be generalizable to countries outside of Iran, as it does not consider cultural and developmental factors.

## Conclusion

Individuals' driving behavior can be linked to other relatively consistent patterns of behavior, such as personality traits. Among the Big Five personality traits, neuroticism was significantly higher in drivers with a history of severe accidents, while conscientiousness was notably lower. The relationship between certain personality traits and risky driving may not be as straightforward as previously thought. In this study, the link between risky driving and openness to experience was not supported, and the association with extroversion appeared to be somewhat contradictory to findings from earlier research. This indicates that assuming the generalizability of personality traits may not always be valid. However, neuroticism is the most widely recognized personality trait that positively correlates with risky driving behavior. Targeted initiatives designed to address emotional instability and stress, key components of neuroticism, may help reduce hazardous driving practices. For example, driver education and training programs could incorporate psychological assessments and stress management techniques. Furthermore, promoting conscientious behaviors through awareness campaigns could significantly enhance road safety. Policymakers and traffic safety officials should consider incorporating personality assessments into driver licensing procedures and tailoring interventions to address the specific psychological profiles that contribute to dangerous driving behaviors within the Iranian context. By aligning traffic safety measures with psychological insights, Iran has the potential to develop more effective and culturally appropriate strategies to reduce accidents and improve overall road safety.

**Acknowledgement**

M.S. and M.A. designed the study. N.C. and M.A. participated in data collection. S.N. and M.S. analyzed and interpreted the data. All author wrote the main manuscript text. Final approval of manuscript by all authors.

**Authors' contributions**

Conceptualization and study design: Dr. Heydari, Dr. Lankarani, Dr. Bazrafshan, and Dr. Sarikhani.

Case collection and data gathering: Mr. Masoumi, Mr. Kargar, Ms. Ghahramani, Ms. Amani, and Ms. Sadeghi. 

Statistical analysis and data interpretation: Dr. Heydari, Dr. Fereidooni, and Dr. Ayatizadeh. Manuscript draft preparation: Dr. Fereidooni, Dr. Sarikhani, and Dr. Ayatizadeh. 

All authors reviewed the results and approved the final version of the manuscript.
